# Intravascular Hemolysis and Septicemia due to *Clostridium perfringens* Emphysematous Cholecystitis and Hepatic Abscesses

**DOI:** 10.1155/2015/523402

**Published:** 2015-07-01

**Authors:** Justin Cochrane, Lacie Bland, Mary Noble

**Affiliations:** Internal Medicine Residency of Spokane, University of Washington Medical School, Spokane, WA 99204, USA

## Abstract

*Context*. *Clostridium perfringens* septicemia is often associated with translocation from the gastrointestinal or gastrourinary tract and occurs in patients who have malignancy or are immunocompromised. *Clostridium perfringens* septicemia is usually fatal without early identification, source control, and antibiotics. *Case*. We present a case of a 65-year-old female with *Clostridium perfringens* septicemia secondary to emphysematous cholecystitis, with progression to hepatic abscesses. *Conclusion*. Septicemia secondary to *Clostridium perfringens* is generally fatal if not detected early. Source control with surgery or percutaneous drainage and early antibiotic therapy is imperative. Hyperbaric oxygen therapy may reduce mortality. Clinicians caring for patients with sepsis and intravascular hemolysis must have *Clostridium perfringens* septicemia on their differential diagnosis with a low threshold for starting antibiotics and pursuing source of infection.

## 1. Introduction

*Clostridium perfringens* is an anaerobic gram positive rod of the human bowel and genital tract.* Clostridium* septicemia occurs mostly in patients with malignancy or an immunocompromised state.* Clostridium* septicemia is usually fatal without early identification, source control, and antibiotics. We present a case of a 65-year-old female with* Clostridium perfringens* septicemia secondary to emphysematous cholecystitis with progression to hepatic abscesses and discuss its relevance to practicing clinicians.

## 2. Case

A 65-year-old female with type 2 diabetes mellitus, coronary artery disease, peripheral arterial disease, and a prior episode of pancreatitis presented with abdominal pain, nausea, and vomiting for 8 hours prior to admission. The abdominal pain was diffuse, cramping, and unrelenting 10 out of 10 severity. She stated that the pain was similar to pain of her prior pancreatitis of unknown etiology 18 months earlier. She had one episode of mild diarrhea with no fever, shakes, or chills prior to admission. Initial exam demonstrated normal vital signs, diffuse abdominal pain to palpation without rebounding our guarding, and negative Murphy's sign. Laboratory evaluation demonstrated lipase 754 U/L (normal level 0–160 U/L), AST 128 IU/L (normal level 0–56 IU/L), ALT 72 IU/L (normal level 7–56 IU/L), and normal complete blood count (CBC) and basic metabolic panel (BMP). Abdominal ultrasound demonstrated no cholecystitis or cholelithiasis and a normal diameter of intrahepatic and extrahepatic bile ducts. Computed tomography (CT) of the abdomen with contrast demonstrated a mild fatty infiltrate of the liver and no gallstone, splenomegaly, or pancreatitis. Pancreatitis was diagnosed, based upon the lipase and abdominal pain, accordant with practice guidelines by the American College of Gastroenterology [1]. Initial treatment consisted of bowel rest, intravenous fluids, and analgesics.

**Figure 1:**
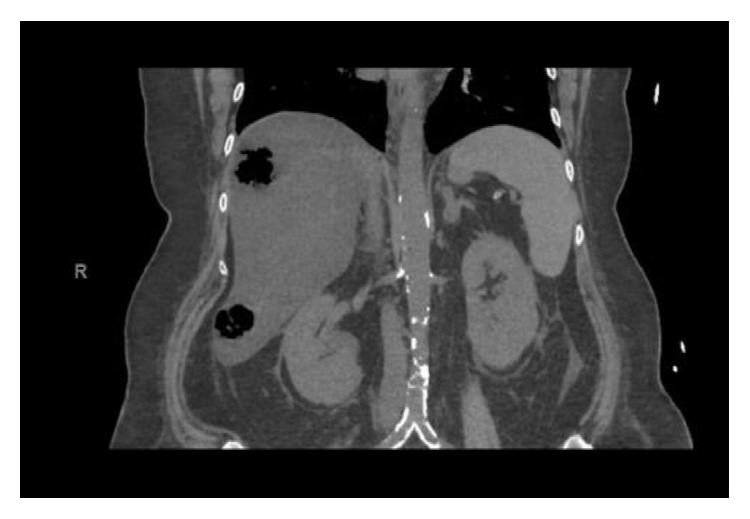
CT abdomen/pelvis demonstrating multiple abscesses in the liver with air fluid levels.

Over the next 36 hours she showed little improvement, developed red tinged urine, and became anemic, and evaluation revealed hemolysis. Hemoglobin declined from 13 to 8.1 g/dL, lactate dehydrogenase was found to be 5290 IU/L (normal level 140 to 280 IU/L), haptoglobin was less than 10 mg/dL (normal level 45 to 165 mg/dL), and fibrinogen and INR were normal. Peripheral smear demonstrated microspherocytes and vacuolated neutrophils with no evidence of schistocytes. Coombs test was negative. White blood cell count increased to 24 K/*μ*L (normal level 4.5 to 10 K/*μ*L) and creatinine increased from 1.0 mg/dL to 2.0 mg/dL. She became hemodynamically unstable with pulse 130 beats per minute, blood pressure 105/50 mmHg, and temperature 103.5 F. Additional testing demonstrated venous lactate 46 mEq/L (normal level 0.5–2.2 mEq/L), ALT 407 IU/L (normal level 7–56 IU/L), AST 1200 IU/L (normal level 0–56 IU/L), ALP 300 IU/L (normal level 44 to 147 IU/L), and total bilirubin 9.6 mg/dL (normal level 0.3 to 1.9 mg/dL). Urine analysis demonstrated 4+ blood on the dipstick with few red blood cells on microscopy. The constellation of hemolysis with sepsis raised the clinical suspicion for* C. perfringens* infection and she was started on high dose Penicillin G 12 million units daily and Clindamycin 900 mg every 8 hours. Hyperbaric oxygen therapy was unavailable. Repeat CT abdomen/pelvis without contrast demonstrated hepatic abscesses (Figure 1).

Percutaneous drains were placed in her hepatic abscesses and gallbladder. Blood cultures drawn prior to antibiotic therapy grew* Clostridium perfringens*. She received 21 days of antibiotics in the hospital and was discharged home and completed 39 more days of high dose Penicillin G. She required a prolonged antibiotic course because repeat CT imaging demonstrated continued hepatic abscesses.

## 3. Discussion

It is critical to review the limited diagnosis of patients with sepsis-like syndrome and hemolysis into infectious and noninfectious etiologies. Infections consist of malaria,* Babesia*,* Bartonella*, and Clostridia and noninfectious include transfusion reaction, glucose-6-phosphate deficiency, and paroxysmal nocturnal hemoglobinuria.

Hematologic studies can help aid in diagnosis of hemolysis associated with* C. perfringens *septicemia including peripheral smear consisting of microspherocytes and vacuolated neutrophils without schistocytes, negative Coombs, and low mean corpuscular volume (MCV) [2]. Our patient demonstrated these peripheral smear characteristics, negative Coombs, and low MCV and with uncontrolled diabetes mellitus seen as a risk factor, empiric antibiotics were started prior to the availability of the culture results demonstrating* C. perfringens.*

*C. perfringens* is normal human flora in the genitourinary and gastrointestinal tract. Immunocompromised states are normally the underlying catalyst allowing* C. perfringens* infections including poorly controlled diabetes mellitus, underlying malignancy ranging from solid tumors to leukemia, and chemotherapy and radiation treatments associated with cancer [3].

Septicemia secondary to* C. perfringens* accounts for only 3% of all positive blood cultures but has a mortality rate of 70% to 100% without prompt diagnosis [4, 5]. Identifying patients at increased risk is crucial. The vast majority of clinical manifestations are often mistaken for more common infections. However, 7% to 15% of patients will develop hemolytic anemia, secondary to phospholipase C lecithinase (alpha toxin) degrading phospholipid in the red cell membranes causing accelerated destruction [6]. Phospholipase C lecithinase may also cause platelet destruction leading to thrombocytopenia. This constellation along with a pigment nephropathy will mimic hemolytic uremic syndrome, but* C. perfringens septicemia *lacks schistocytes in the peripheral smear.

Source control is imperative in patients with septicemia secondary to* C. perfringens.* Delayed identification leads to a mortality rate of 70% to 90% with average time to mortality of 9.7 hours [2]. Source control with surgical debridement or percutaneous drainage improves mortality to 25% to 30% [2]. Additional survival benefit has been demonstrated in case series for adjuvant hyperbaric oxygen therapy (HBOT), as higher tissues oxygen concentration inhibits Clostridia toxin production, destroys bacteria, and inactivates existing toxins [2, 3]. van Bunderen et al. [6] reviewed cases of septicemia secondary to* C. perfringens* in the literature from 1990 to 2012 with the majority of sources being genitourinary or gastrointestinal in origin. Shindo et al. [7] demonstrated similar findings in a case series of 33 patients over a 13 year time period, with 27% of patients having involvement of the biliary tract. Our patient's source was found to be secondary to emphysematous cholecystitis (EC), a form of acute cholecystitis, of which 50% of cases are* C. perfringens*. Her case is unique in that she originally presented with cholestasis without evidence of gallstone or choledocholithiasis which progressed quickly to cholangitis causing EC, progressing to hepatic abscesses.

Early initiation of antibiotics is crucial. High dose Penicillin G of 10 to 24 million units daily and Clindamycin 2,700 mg daily is the standard for* C. perfringens*. Clindamycin inhibits production of virulent proteins, such as phospholipase C lecithinase. The inhibition of toxin production by Clindamycin will reduce the probability of developing worsening hemolysis and renal failure as is theoretically seen in monotherapy with high dose Penicillin G. The combination of high dose Penicillin G and Clindamycin demonstrates reduced mortality regardless of age, comorbid conditions, or presence of hemolysis on admission [2]. Standard treatment duration consists of high dose Penicillin G and Clindamycin for 10–14 days, but all patients need antibiotic therapy duration tailored based on improvement of infection through clinical and radiographic resolution [8, 9].

## 4. Conclusion

Septicemia secondary to* Clostridium perfringens* is fatal if not detected early. Source control with surgery or percutaneous drainage and early antibiotic therapy with high dose Penicillin G and Clindamycin to decrease toxin production is imperative. Adjuvant HBOT has also shown survival benefit and should be considered if available. Clinicians caring for a patient with sepsis and intravascular hemolysis must suspect* Clostridium perfringens* septicemia.

## Appendix

See Figure 1.
